# Aplicación de la guía de trauma dental de la asociación internacional de traumatología dental en un grupo de odontólogos paraguayos

**DOI:** 10.21142/2523-2754-0904-2021-083

**Published:** 2021-12-09

**Authors:** José Gamarra, Osmar Gómez, Cecilia Olmedo, Stela Benítez de Forcadell, Clarisse Díaz-Reissner, Vicente Fretes

**Affiliations:** 1 Facultad de Odontología de la Universidad Nacional de Asunción. San Lorenzo, Paraguay. josemgamarra31@gmail.com, oagi5897@gmail.com, ceciolmedo@gmail.com, stelamary@hotmail.es, cdiazr@odo.una.py, vicentefretes@odo.una.py Universidad Nacional de Asunción Facultad de Odontología Universidad Nacional de Asunción San Lorenzo Paraguay josemgamarra31@gmail.com oagi5897@gmail.com ceciolmedo@gmail.com stelamary@hotmail.es, cdiazr@odo.una.py vicentefretes@odo.una.py

**Keywords:** traumatismos de los dientes, encuestas y cuestionarios, conocimiento, tooth injuries, surveys and questionnaires, knowledge

## Abstract

**Objetivo:**

Evaluar la aplicación de la guía de trauma dental según la IADT en un grupo de odontólogos paraguayos entre los meses de enero y mayo del 2021. El estudio fue observacional descriptivo de corte transversal. Formaron parte del estudio un grupo de odontólogos paraguayos con acceso a internet que deseaban participar. El instrumento de medición utilizado fue un cuestionario cerrado de 20 preguntas basadas en la guía de la IADT, confeccionado en formularios de Google, distribuidos por mensajería instantánea entre los meses de enero y abril del 2021. Basado en la Guía para la Evaluación y Manejo de Lesiones Dentales Traumáticas de la Asociación Internacional de Traumatología Dental (IADT), el cuestionario cuenta con 20 preguntas sobre la evaluación de signos clínicos y radiográficos, determinación de variables de tratamiento, diferenciación del tipo de tratamiento, consecuencias biológicas del trauma, materiales y tiempo de entablillado, e indicaciones postoperatorias y medicación.

**Resultados:**

Participaron 230 odontólogos, con un promedio de 33 (DE = 6,4) años y el 70,43% fueron mujeres. El 30,87% fueron odontólogos generales y el 70,87% ejerció exclusivamente en consultorio privado. El 68,70% respondió que poseía experiencia acerca del manejo y tratamiento de traumatismos dentoalveolares. El 70,87% no ha escuchado hablar o leído acerca de la guía de trauma dental de la IADT. En cuanto al nivel de conocimiento, el 60% obtuvo medio.

**Conclusión:**

La mayoría de los odontólogos encuestados tuvo conocimiento medio, por lo que se recomienda promover el conocimiento de las guías internacionales de tratamiento de TDA.

## Abstract (en)

**Objective:**

To evaluate the application of the dental trauma guide according to the International Association of Dental Traumatology in a group of Paraguayan dentists from April to May 2021. The was a descriptive, cross-sectional, observational study. A group of Paraguayan dentists with internet access accepted to participate in the study. The measurement instrument was a closed questionnaire from Google Forms, distributed by instant messaging (WhatsApp) from January to April 2021. Based on the Guidelines for the Evaluation and Management of Traumatic Dental Injuries by the International Association of Dental Traumatology (IADT), the questionnaire includes 20 questions on the assessment of clinical and radiographic signs, determination of treatment variables, differentiation of the type of treatment, biological consequences of trauma, materials and splint time, and postoperative indications and medication.

**Results:**

230 dentists participated; the mean age was 33 (SD = 6.4) years, 70.43% were women, 30.87% were general dentists, and 70.87% were practicing exclusively in private practice. 68.70% responded that they were familiar with the management and treatment of dentoalveolar trauma, while 70.87% had not heard or read about the IADT dental trauma guide. Regarding the level of knowledge, 60.00% scored an average mark.

**Conclusion:**

Most of the dentists surveyed had moderate knowledge regarding the evaluation and management of traumatic dental injuries.

## INTRODUCCIÓN

Un traumatismo dentoalveolar (TDA) se puede generar a partir de un impacto agresivo sobre estructuras dentales y tejidos adyacentes, que derivan en algún tipo de lesión. Pueden ser afectadas las piezas dentarias, la articulación temporomandibular y el tejido óseo, así como los tejidos blandos circundantes de encías, labios, mejillas y piso de la boca ^1^, lo que genera secuelas tanto fisiológicas como estéticas. Según reportes del año 1999, en países como el Reino Unido, la frecuencia de TDA fue del 58,6%, mientras que en Chile, en el 2010, se reportó una prevalencia del 58,77% ^2^.Se debe tener en cuenta que, en Chile, la mayor incidencia de TDA se dio en niños con dentición primaria y mixta, entre los 2 y 4 años, y los 8 y 10 años, respectivamente ^3^. Debido a que la práctica de deporte es más frecuente en estos grupos etarios ^4^, las caídas de su propia altura son la principal etiología, generalmente en varones ^5^^,^^6^, y presenta mayor gravedad en aquellos que tenían relación labial incompetente y resalte aumentado ^7^ en estudios realizados en Cuba.

Por otra parte, las lesiones traumáticas dentales requieren de la habilidad del profesional, por la magnitud del problema y la fragilidad emocional de los niños. El odontólogo debe estar capacitado para atender este tipo de eventos ^8^, ya que debe realizar un tratamiento inmediato de urgencia y un seguimiento a través del tiempo. Es necesaria una visión de rehabilitación integral resumida en la aplicación de protocolos aplicados a las necesidades y al momento en el que se encuentra el paciente con el objetivo de devolver forma, función y estética ^9^. Para el buen pronóstico de los dientes traumatizados, es de vital importancia la rapidez y la efectividad con que la lesión es tratada, luego del accidente ^10^.

Cuando un odontólogo se enfrenta a un paciente que ha sufrido un trauma dentoalveolar, debe ser capaz de identificar grado de lesión, lugar del hecho, grado de contaminación de la pieza, medios de conservación de la pieza fracturada y medidas para resolver la situación presentada ^11^. Además, se recomienda crear conciencia en padres, representantes, educadores y profesionales de la salud sobre la importancia de recurrir al odontólogo en caso de ocurrir estos eventos y no solamente cuando existe dolor o se ha perdido la estética ^10^. Actualmente, la Organization Dental Trauma Guide posee un sitio web desarrollado en cooperación con el Hospital Universitario de Copenhague y la Asociación Internacional de Traumatología Dental (IADT), creada con el fin de proveer una guía sistemática sobre protocolos para resolver distintos tipos de lesiones dentoalveolares ^12^^-^^14^.

Si bien no existen estudios sobre la prevalencia en Paraguay, los TDA en el campo de la odontología en nuestro país siguen siendo un tema de suma importancia. De no abordarse satisfactoriamente, podrían generar la pérdida dentaria y repercutir en la autoestima del niño o adolescente. Debido a la frecuencia de las TDA en esta población, el odontólogo que trabaja tanto en el sector público como privado debe estar familiarizado con los protocolos de atención. 

Con el presente trabajo, se pretende dar a conocer pautas en cuanto al protocolo sobre el manejo de TDA basados en la odontología basada en evidencia que establecen un protocolo de acción, para así compararlos con las prácticas clínicas que manejan los profesionales paraguayos. Esto permitirá establecer diferencias o similitudes entre el manejo que se da en la práctica clínica y las recomendaciones de protocolos internacionales, lo que evidencia las falencias o limitaciones, en caso las hubiere, que posee el odontólogo del área de salud pública sobre el manejo de TDA. En la práctica clínica, dentro del ámbito estudiantil de grado, es muy poco frecuente el tratamiento de traumatismos. Estos resultados también podrán ser útiles para replantear su importancia y la necesidad de incorporar la enseñanza con mayor énfasis en el curricular de la carrera.

Por lo expuesto, se planteó como objetivo del estudio evaluar la aplicación de la guía de trauma dental, según la Asociación Internacional de Traumatología Dental, en un grupo de odontólogos paraguayos entre los meses de enero y mayo del 2021.

## MATERIALES Y MÉTODOS

El estudio es observacional descriptivo de corte transversal. Formaron parte del estudio un grupo de odontólogos paraguayos con acceso a internet que deseaban participar. El protocolo de investigación fue aprobado por el Comité de Ética en Investigación de la Facultad de Odontología de la Universidad Nacional de Asunción.

El instrumento de medición utilizado fue un cuestionario cerrado confeccionado en formularios de Google, distribuidos por mensajería instantánea (WhatsApp) entre los meses de enero y mayo del 2021. Este se basó en la guía sobre el manejo de los traumatismos dentoalveolares confeccionado por la International Association of Dental Traumatology (IADT). Cuenta con una serie de preguntas numeradas del 1 al 20. A través del mismo, se obtuvieron datos demográficos y profesionales acerca del manejo de traumatismo, y se hicieron preguntas sobre la valoración signos clínicos y radiográficos (ítem 20), la determinación de variables de tratamientos (ítems 5, 7, 8 y 16), la diferenciación del tipo de tratamiento (ítems 1, 2, 3, 4, 6, 10, 18), las consecuencias biológicas de los traumatismos (ítems 9, 13 y 15), los materiales y tiempo de ferulización (ítems 11 y 14), y las indicaciones posoperatorias y la medicación (ítems 15 y 17).

Cada pregunta contó con una única respuesta correcta, siendo computadas como correcta e incorrecta, basadas en la guía sobre el manejo de los traumatismos dentoalveolares confeccionado por la IADT. Por tanto, el nivel de aplicación es el siguiente: alto (del 90 al 100%, de 18 a 20 puntos), medio (del 70 al 80%, de 13 a 18 puntos) y bajo (del 0 al 60%, de 0 a 12 puntos).

Para la elaboración del instrumento de medición y las recomendaciones difundidas, se tomó como referencia el trabajo de investigación realizado en Brasil sobre el conocimiento de los odontólogos sobre el trauma dental ^15^, el realizado en Croacia acerca de los conocimientos sobre las prácticas de emergencia en casos de trauma dental en niños ^16^, y la Guía de Trauma Dental desarrollada por la Asociación Internacional de Traumatología Dental ^12^^-^^14^. Se llevó a cabo una prueba piloto con 3 profesionales, en la que el equipo de investigación unificó directrices y ajustó el instrumento de medición.

Se realizó el cálculo del tamaño de la muestra para una población finita a fin de estimar una proporción desconocida tomando como referencia el 50% de nivel de conocimiento medio y alto, con una precisión del 7% y un nivel de confianza del 95%. Se sugiere a encuestar a 196 odontólogos como mínimo, pero, considerando la pérdida de datos del 15%, sería mejor encuestar a 218 odontólogos para obtener una muestra más representativa. El muestreo se realizó por conveniencia.

Los datos fueron analizados con el programa Epi Info 7.2.4. Se utilizaron estadísticas descriptivas utilizando frecuencia y porcentaje, presentados en tablas y gráficos para las variables cualitativas, mientras que, para las variables cuantitativas, se analizarán a través del promedio y desvío estándar.

## RESULTADOS

Participaron del estudio 230 odontólogos, con una edad promedio de 33 años (DE = 6,4). El 70,43% fueron mujeres; el 30,87%, odontólogos generales; el 69,1%, odontólogos especialistas, y el 70,87% ejerce exclusivamente en un consultorio privado (Tabla 1).

**Tabla 1:** Odontólogos encuestados según variables demográficas y profesionales

Variable	N	%
Sexo		
Femenino	162	70,43
Masculino	68	29,57
Especialidad		
General	71	30,87
Endodoncia	42	18,26
Rehabilitación	32	13,91
Cirugía oral	20	8,70
Odontopediatría	16	6,96
Implante	14	6,09
Ortodoncia	13	5,65
Operatoria	12	5,22
Periodoncia	8	3,48
Cirugía maxilofacial	2	0,87
Años de ejercicio de la profesión		
1 a 3	1	0,43
4 a 5	96	41,74
6 a 9	82	35,65
Mayor a 10	51	22,17
Sector que ejerce la profesión		
Exclusiva docencia	2	0,87
Exclusivo privada	163	70,87
Exclusivo pública	4	1,74
Pública y privada	61	26,52

El 68,70% de los encuestados respondió que posee experiencia en el manejo y tratamiento de traumatismos dentoalveolares. El 70,87% no ha escuchado hablar ni ha leído acerca de la guía de trauma dental de la IADT.

El instrumento de medición fue un cuestionario elaborado siguiendo directrices de la IADT establecidos en su guía de trauma dental, mediante preguntas simples de baja complejidad (tratamiento de fracturas de esmalte, dentina, coronoradicular, elementos para ferulizar) (Tabla 2) y preguntas más complejas (tratamiento de avulsión, luxaciones, esquema de vacunación, cuidados posoperatorios) (Tabla 3).

**Tabla 2:** Respuestas al cuestionario

N.o	Pregunta	N	%
1	Al presentarse una factura de esmalte con pérdida de estructura dentaria sin exposición de dentina, el mejor tratamiento que usted emplearía es…		
	Restauración con resina compuesta	219	95,22
	Restauración con ionómero de vidrio	2	0,87
	Restauración con resina fluida	9	3,91
	No sabe.	0	0,00
2	Un paciente sufre una fractura de esmalte con pérdida de estructura dentaria sin exposición de dentina, se presenta a la consulta con el fragmento en mano. Según su experiencia, ¿cómo la trataría?		
	Restauración con resina compuesta	31	13,48
	Restauración con ionómero de vidrio	1	0,43
	Reposicionar el fragmento mediante *collage* dentario	197	85,65
	No sabe.	1	0,43
3	Un paciente acude con fractura del 2,1 con una pequeña exposición pulpar. El tratamiento de elección acorde a la situación clínica del paciente sería…		
	Tratamiento de conducto de la pieza afectada	28	12,17
	Protección pulpar directa o pulpotomía parcial	63	27,39
	Solo protección pulpar directa y control radiográfico	138	60,00
	No sabe.	1	0,43
4	El tratamiento que usted aplicará a un diente con ápice cerrado que sufrió una fractura corono-radicular con evidente exposición pulpar es…		
	Retiro del fragmento de la corona, tratamiento endodóntico y restauración	179	77,83
	Exodoncia de la pieza dentaria	12	5,22
	Tratamiento endodóntico de la pieza sin restauración definitiva	38	16,52
	No sabe.	1	0,43
5	En el caso de una concusión, ¿qué tipos de tratamientos usted cree que podemos realizar?		
	Ferulización del traumatismo + endodoncia	8	3,48
	Ferulización + seguimiento clínico y radiográfico	97	42,17
	No se necesita tratamiento, solo seguimiento radiográfico al año	114	49,57
	No sabe.	11	4,78
6	El tratamiento de elección que se debe seguir en los casos de una fractura radicular, específicamente en su tercio medio, según su experiencia, es…		
	Exodoncia de la pieza dentaria en cuestión	85	36,96
	Reposición del fragmento si lo hay, ferulizar por 4 semanas	125	54,35
	Tratamiento endodóntico	19	8,26
	No sabe.	1	0,43
7	¿En qué lesión traumática dentoalveolar no indicaría como tratamiento una estabilización o ferulización con alambre de tipo flexible?		
	Luxación extrusiva	27	11,74
	Avulsión dentaria	31	13,48
	Concusión	172	74,78
	No sabe.	0	0,00
8	Un incisivo central superior derecho con movilidad clínica evidente. En la radiografía, revela una raíz madura con una fractura en el tercio medio de la raíz. La lesión ocurrió un día antes. Ante los antecedentes, ¿qué tratamiento se indica?		
	Solamente medicación	10	4,35
	Monitoreo clínico y radiográfico del diente	98	42,61
	Ferulización y tratamiento endodóntico	119	51,74
	No sabe.	3	1,30
9	En las lesiones como las luxaciones laterales, si la pulpa se vuelve necrótica, el tratamiento ideal es la endodoncia para prevenir algunas patologías como…		
	Inflamación del tejido periapical	39	16,96
	Reabsorción radicular	152	66,09
	Infección radicular	39	16,96
	No sabe.	0	0,00
10	Un diente inmaduro (formación radicular incompleta) presenta luxación intrusiva el protocolo a seguir para el tratamiento es…			
	Permitir la erupción sin intervención	203	88,26
	Reubicación ortodóncica	22	9,57
	Exodoncia de la pieza dentaria	4	1,74
	No sabe.	1	0,43

**Tabla 3:** Respuestas al cuestionario (continuación)

N.o	Pregunta	N	%
11	En los casos de los traumatismos en los que se debe realizar una ferulización, los materiales que utiliza son…		
	Alambre flexible + resina	205	89,13
	*Brackets* de ortodoncia + resina	0	0,00
	Resina composite	14	6,09
	No sabe.	11	4,78
12	Las dos condiciones esenciales para que el reimplante de un diente avulsionado sea efectivo son…		0,00
	Lugar del accidente + tiempo transcurrido	79	34,35
	Lugar de accidente + condición de las células del LP	113	49,13
	Condición de las células del LP + tiempo transcurrido	38	16,52
	No sabe.	0	0,00
13	El tiempo transcurrido para la reimplantación de un diente avulsionado y mantenido en condiciones seca es…		
	No se debe reimplantar si ha estado seco.	23	10,00
	Entre 30 y 60 minutos como máximo	44	19,13
	Lo antes posible, menor a 30 minutos postrauma	162	70,43
	No sabe.	1	0,43
14	Para que un diente avulsionado se encuentre en condiciones favorables para la realización del tratamiento de conducto, ¿cuánto tiempo debe estar ferulizado?			
	1 mes	46	20,00
	2 semanas	120	52,17
	3 meses	64	27,83
	No sabe.	0	0,00
15	¿Qué esquema de vacunación y que vía de administración de antibiótico le indicaría a un paciente que ha sufrido avulsión en condiciones no favorables (diente contaminado)?			
	DTPa + administración de ATB vía oral	169	73,48
	Vacuna triple vírica + administración de ATB vía tópica	30	13,04
	Hepatitis B + administración de ATB vía tópica	31	13,48
	No sabe.	0	0,00
16	Se presenta un niño a consulta con un diente con ápice abierto que ha sufrido una avulsión, el diente ha sido reimplantado en el momento por sus allegados en el lugar del accidente, el protocolo que realiza como atención de urgencia es…			
	Examen radiográfico + tratamiento endodóntico inmediato	29	12,61
	Examen radiográfico y clínico + posterior ferulización + control evolutivo	194	84,35
	Solo un control de dieta, porque el diente ya se reimplantó.	7	3,04
	No sabe.		0,00
17	Las indicaciones posoperatorias ideales que se indica a un paciente que ha sufrido una avulsión		0,00
	Dieta blanda por 1 mes + evitar la zona traumatizada durante la higiene	28	12,17
	Dieta blanda hasta 2 semanas + lavar los dientes con cepillo suave	161	70,00
	Dieta líquida, evitar todo tipo de deporte por 1 mes e higienizar solo con colutorio	37	16,09
	No sabe.	4	1,74
18	En un diente avulsionado con ápice cerrado que ha sido reimplantado dentro de los 30 minutos postrauma en el sitio del accidente, se indica…		
	Tratamiento mediato del conducto radicular + ferulización	65	28,26
	Medicación + ferulización de la pieza afectada	157	68,26
	Evitar la reimplantación	7	3,04
	No sabe.	1	0,43
19	En el examen radiográfico de la luxación extrusiva, sin presencia de fractura alveolar, 6 horas postrauma, se visualiza…		0,00
	Agrandamiento del ligamento periodontal	120	52,17
	Ubicación del trazo de fractura (tercio coronal, medio o apical)	85	36,96
	Extensión apical de la línea de la fractura	25	10,87
	No sabe.	0	0,00
20	¿Cuáles son los signos y síntomas que se deben tener en cuenta tras realizar el tratamiento de una fractura alveolar para identificar su éxito, teniendo en cuenta un control a los 3 meses después de haberlo tratado?		
	Respuesta pulpar positiva (vitalidad pulpar) sin señal de periodontitis apical	126	54,78
	Movilidad del fragmento alveolar fracturado	91	39,57
	Dolor en la masticación	12	5,22
	No sabe.	1	0,43

Se observó un mayor número de respuestas correctas (Tabla 2) en ítems que hacen referencia a tratamientos restauradores, con resina compuesta o técnicas de restauración en casos de fractura de esmalte con pérdida o no del fragmento dentario, de baja complejidad, como la pregunta 1 (95%) y la pregunta 2 (85%). Lo mismo ocurrió con la pregunta 10, que se refiere a los tratamientos paliativos para una luxación intrusiva (88%). 

En la elección del tipo de material ideal que se utiliza para la ferulización (pregunta 11) (Tabla 3), un 89,13% respondió correctamente; así como un 73,13% conocía el esquema de vacunación profiláctica y la terapia medicamentosa básica indicada en los casos de traumatismos complejos como la avulsión (pregunta 15). En el caso de la pregunta 16, el 84,35% manejaría de manera ideal el protocolo de atención de urgencia con antecedente de avulsión y reimplantación de la pieza dentaria en el lugar, ideal para el éxito en el tratamiento. En donde se evidenció una menor tasa de respuesta correcta fue en la pregunta 12, que hace referencia a las condiciones esenciales para un reimplante exitoso en una avulsión, pues solo el 16,52% de los encuestados respondió correctamente. 

**Figura 1:**
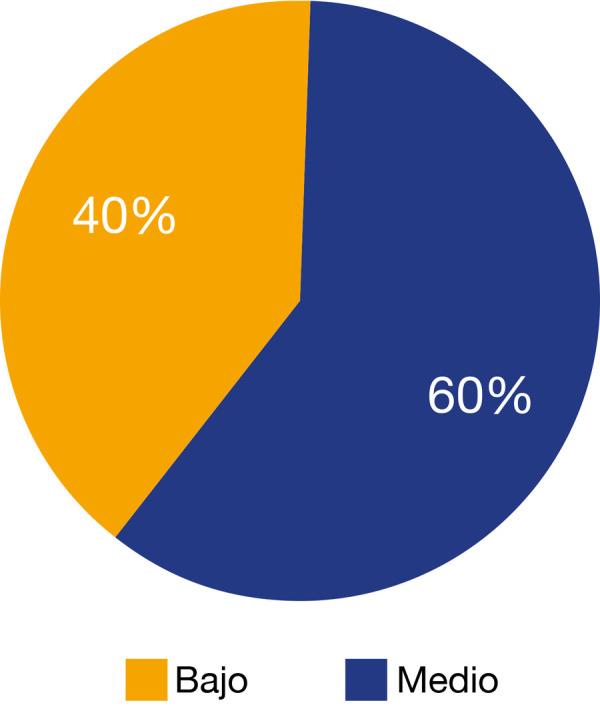
Nivel de aplicación de la guía de trauma dental de la IADT

En cuanto al nivel de conocimiento, el 60% obtuvo nivel medio, ningún odontólogo obtuvo un nivel alto (Figura 1).

## DISCUSIÓN

El presente estudio tuvo como objetivo medir el nivel de aplicación de la guía de trauma dental según la IADT en el tratamiento de los TDA en un grupo de odontólogos. Se basó en la aplicación de un cuestionario que recolectó datos sociodemográficos como sexo, especialidad, años de ejercicio en la profesión y preguntas específicas relacionadas con el tratamiento de los diversos tipos de traumatismos dentoalveolares. El conocimiento general en los odontólogos encuestados fue medio.

Del total de los encuestados, un 30,87% fueron odontólogos generales y un 69,1% poseía un título de posgraduación en alguna rama de la odontología, cifras superiores a las encontradas en un estudio realizado en Líbano, con solo un 8% de encuestados especialistas ^17^. Esto podría deberse a que la mayoría de los profesionales en nuestro país opta por seguir formándose en especialidades odontológicas.

Respecto de si poseen experiencia previa en el tratamiento de los traumatismos dentoalveolares, un 68,70% de los encuestados respondió que sí la posee, porcentaje más elevado en comparación con los estudios realizados en Turquía y Líbano, donde se obtuvo un 55,5% y un 36,8%, respectivamente ^(17, 18)^. Esto podría deberse a que en otros países son directamente derivados a especialistas, mientras que en nuestro país el odontólogo general atiende en el consultorio este caso de urgencia en primera instancia, para luego derivarlo al endodoncista si el caso lo requiere.

El 70,87% no ha escuchado hablar o leído acerca de la guía de trauma dental de la IADT. El idioma podría ser una barrera fundamental para la poca utilización y fomento de la guía en los países de Latinoamérica ^19^. Debe tenerse en cuenta que estas guías, generalmente, no forman parte del programa en el grado y solamente algunos posgrados de endodoncia la comparten con sus estudiantes, motivo por el cual no son tan difundidas ni conocidas.

En los casos de fractura de esmalte y dentina sin comprometimiento pulpar, el tratamiento de elección según la IADT es la restauración, si no existe el fragmento dentario fracturado, con resina compuesta ^12^. Un 95% de los encuestados respondió de manera correcta en el tratamiento de este tipo, dato que no coincide con el estudio de Líbano, en el que un 88,9% de los encuestados colocaría primero una capa de hidróxido de calcio y solo un 3,5% realizaría la restauración con resina compuesta ^17^^,^^20^. 

Sobre la consecuencia biológica esperada como resultado de una luxación lateral, la reabsorción radicular ^12^, el 66,09% indicó la respuesta correcta, dato que no coincide con los datos proporcionados por encuestados en un estudio en Alemania, donde más de la mitad consideraba que sería una infección radicular, lo que coincide con otro estudio realizado en Turquía ^18^^,^^21^. 

Un dato llamativo fueron las respuestas sobre el tratamiento de las fracturas radiculares horizontales, para el que se indica la realización de una férula ^12^. El 54,3% de los encuestados de este estudio coincidió en que se debe ferulizar, pero un 36,96% sigue considerando como tratamiento la exodoncia de la pieza dentaria, dato que coincide con el estudio de Líbano, donde un 55,57% consideró este último tratamiento ^17^. 

En cuanto al tratamiento de la avulsión dental, el tiempo ideal para la reimplantación de la pieza dentaria es lo antes posible y solo se procede a la reposición en piezas dentarias de la dentición permanente. Si la reimplantación se realizó dentro de un tiempo prudente, se sigue el protocolo de reposición, control radiográfico, estabilización, dieta y posterior tratamiento endodóntico ^13^^,^^14^. En el presente estudio, un 84,35% respondió de manera correcta acerca del protocolo que se debe realizar una vez reimplantada la pieza dentaria en un tiempo prudente, dato que coincide con el estudio hecho en Líbano, en el cual un 88% de los encuestados respondió de la misma manera. Si bien concuerda con este estudio, no lo hace con otro realizado en Santa Catarina (Brasil), en el que solo el 37,1% mencionó que se debe realizar lo antes posible ^22^. Con respecto al tiempo, el 70,43% respondió que la reimplantación se debe realizar lo antes posible, dato que coincide con el estudio realizado en la India, en el que el 56,8% sugirió lo mismo, y con el estudio realizado en Santa Catarina ^23^^,^^24^. 

En cuanto a la medicación, la guía hace referencia al empleo de antibióticos y al esquema de vacunación antitetánica (vacuna DPD) ^13^. El 73,48% de los encuestados coincide en prescribir antibióticos y dicho esquema de vacunación, datos que coinciden en parte con el estudio de Santa Catarina, en el cual el 86% indica terapia antibiótica después de la reimplantación, pero no menciona el esquema de vacunación. Lo contrario ocurre en el estudio de la India, en el que la mayoría de los encuestados indicó la aplicación de la vacuna antitetánica, pero no mencionó terapia antibiótica ^22^^,^^23^. 

El estudio arrojó un nivel de conocimiento medio teniendo en cuenta cómo se aplican los tratamientos descritos en la guía de trauma dental, lo que coincide con varios estudios cuya población de estudio también obtuvo un nivel de conocimiento medio ^15^^,^^22^^,^^25^^,^^26^. Se debe destacar que una comparación entre diferentes investigaciones resulta difícil debido a las diferencias en su metodología y que, a largo plazo, se deberían considerar diseños de estudios estandarizados o investigaciones de encuestas internacionales ^27^^,^^28^. El conocimiento adecuado del profesional odontólogo es fundamental para la resolución adecuada de los traumatismos, por lo que mejorar estos conocimientos debe ser obligatorio, ya que presentan un desafío en la odontología y presentan complicaciones para los pacientes que pueden dar lugar a tratamientos costosos y prolongados ^21^^,^^29^^,^^30^. Si bien la mayoría de los odontólogos encuestados obtuvo un nivel de conocimiento medio y teniendo en cuenta las limitaciones, este estudio brinda elementos para identificar algunos puntos claves que deben ser reforzados en cuanto al conocimiento sobre la aplicación de tratamientos para los traumatismos.

Si bien en los programas de grado se abarca con bastante detalle el estudio de las causas y tratamientos de los diferentes tipos de TDA, al ser un procedimiento que el odontólogo general no realizada de forma rutinaria, es necesaria la utilización de guías que sean accesibles para la consulta y realización de tratamientos adecuados, según las necesidades del caso. Se recomienda, como en otros países, que las autoridades pertinentes, ya sean del Círculo de Odontólogos del Paraguay (COP), las facultades de Odontología y los institutos de posgrado, brinden talleres, cursos o *workshop* sobre el tratamiento de los TDA.

## CONCLUSIÓN

El nivel de conocimiento sobre protocolos de atención a pacientes con traumatismos dentoalveolares en los odontólogos encuestados fue medio, por lo que se recomienda promover el conocimiento de las guías internacionales de atención a pacientes con estos traumatismos.

## Fuente de financiamiento:

Autofinanciado
